# Role of the Ion Channel Extracellular Collar in AMPA Receptor Gating

**DOI:** 10.1038/s41598-017-01146-z

**Published:** 2017-04-21

**Authors:** Maria V. Yelshanskaya, Samaneh Mesbahi-Vasey, Maria G. Kurnikova, Alexander I. Sobolevsky

**Affiliations:** 1 Department of Biochemistry and Molecular Biophysics, Columbia University, 650 West 168th Street, New York, NY 10032 USA; 2 Chemistry Department, Carnegie Mellon University, 4400 Fifth Ave, Pittsburgh, PA 15213 USA

## Abstract

AMPA subtype ionotropic glutamate receptors mediate fast excitatory neurotransmission and are implicated in numerous neurological diseases. Ionic currents through AMPA receptor channels can be allosterically regulated via different sites on the receptor protein. We used site-directed mutagenesis and patch-clamp recordings to probe the ion channel extracellular collar, the binding region for noncompetitive allosteric inhibitors. We found position and substitution-dependent effects for introduced mutations at this region on AMPA receptor gating. The results of mutagenesis suggested that the transmembrane domains M1, M3 and M4, which contribute to the ion channel extracellular collar, undergo significant relative displacement during gating. We used molecular dynamics simulations to predict an AMPA receptor open state structure and rationalize the results of mutagenesis. We conclude that the ion channel extracellular collar plays a distinct role in gating and represents a hub for powerful allosteric modulation of AMPA receptor function that can be used for developing novel therapeutics.

## Introduction

Ionotropic glutamate receptors (iGluRs) are a family of tetrameric ligand-gated ion channels that are critical for central nervous system development and function. They mediate the majority of excitatory neurotransmission and their dysfunction is associated with numerous neurological diseases^1–3^. There are three major iGluR subtypes – NMDA, AMPA and kainate receptors – that have distinct biophysical and pharmacological properties but share a conserved modular design, which comprises two amino-terminal domain (ATD) dimers, two ligand-binding domain (LBD) dimers, transmembrane domains (TMDs) and largely unstructured carboxyl-terminal domains (CTDs). TMDs of the four iGluR subunits, each containing transmembrane helices M1, M3 and M4 and a re-entrant loop, M2, form a cation-selective ion channel. The channel opens or closes for ion conduction in the process termed gating. iGluR gating initiates with agonist binding to the LBD and continues as conformational changes that propagate from the LBD to the ion channel via the LBD-TMD linkers^4^. The two major iGluR gating processes are activation and desensitization. Activation leads to ion channel opening in response to agonist binding, while desensitization results in ion channel closure in the presence of an agonist bound to the receptor.

Structural studies of isolated LBDs that have been crystallized in complex with numerous ligands and uncovered a diverse ensemble of gating conformations^5–9^, greatly facilitating our understanding of the molecular basis of gating initiation. This conformational ensemble was analysed using mutagenesis, various biophysical techniques and theoretical modelling to develop molecular models of gating at the level of LBD^10–25^. In contrast, the available structures of intact receptors in complex with different ligands^26–30^ revealed the ion channel in nearly identical non-conducting conformations. While structural information on AMPA receptor ion channel conformational dynamics remains limited, mutagenesis and functional recordings represent important tools to study molecular bases of gating at the level of ion channel and LBD-TMD linkers. In fact, previous mutagenesis studies identified several domain regions involved in AMPA receptor gating, including the pore-forming portion of M3^31–33^ that comprises the Lurcher site^34^, the “ER” site in the M3-S2 linker^35^ and the “hydrophobic box”, located at the extracellular interface of the transmembrane helices^36^. In the absence of high resolution structural information on the various conformational states of the TMD and LBD-TMD linkers, molecular modelling driven by low resolution information obtained from mutagenesis is an essential tool that is capable of developing instructive and testable models of structures in different conformations^37–39^.

Our recent study of the allosteric mechanism of AMPA receptor noncompetitive inhibition by antiepileptic drugs pyridone perampanel (PMP)^40–42^, GYKI 53655 (GYKI)^43, 44^ and CP 465022 (CP)^44–46^ identified novel antagonist binding sites in the ion channel extracellular collar, at the interface between TMD and LBD-TMD linkers^47^. We hypothesized that these inhibitors stabilize the AMPA receptor in the closed state and act as wedges between transmembrane segments, thereby preventing gating rearrangements necessary for ion channel opening. If our hypothesis is correct, protein mutagenesis in the vicinity of the noncompetitive inhibitor binding sites may have a strong influence on AMPA receptor gating. Supporting this idea, desensitization in the highly homologous and structurally similar NMDA receptors was greatly affected by mutations in a “hydrophobic box”^36^, a region that in AMPA receptors is adjacent to the noncompetitive inhibitor binding sites. To probe the role of the ion channel extracellular collar in gating, we mutated the residues contributing to or adjacent to the noncompetitive inhibitor binding sites. We found several mutations that strongly affected AMPA receptor desensitization and deactivation. Using the mutations that promote ion channel opening or inhibit receptor desensitization, we performed targeted molecular dynamics (MD)^48^ simulations of the TMD and LBD-TMD linkers in lipid membrane and water (full atomistic model) environments to predict an AMPA receptor open state structure. We verified this structure by designing a crosslink that inhibits ion channel opening between the pre-M1 and M4 regions of the collar. Comparing the modelled open state and experimental apo state structures, we rationalized the results of our mutagenesis experiments and predicted gating-related conformational rearrangements in the TMD, including relative displacement of the pre-M1, M3 and M4 segments that contribute to the noncompetitive inhibitor binding sites.

## Results

**Figure 1:**
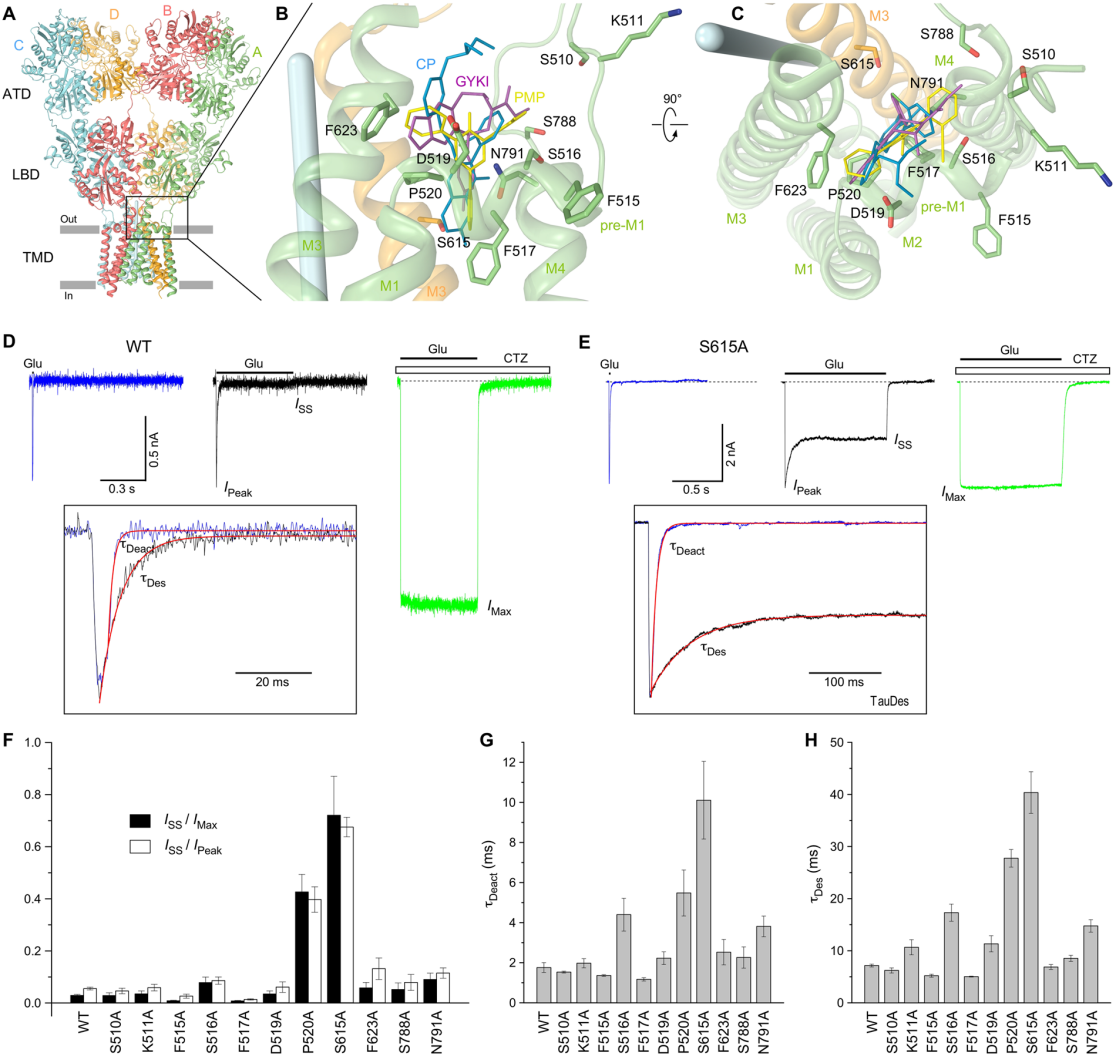
Alanine mutagenesis at the ion channel extracellular collar of GluA2 receptor. (**A**) GluA2_apo_ structure (PDB: 5L1B) viewed parallel to the membrane and perpendicular to the overall two-fold axis of molecular symmetry. Each subunit is in different color. Inner and outer sides of the membrane are indicated by parallel grey bars. (**B**,**C**) Close-up views of the region boxed in (**A**) and comprising noncompetitive inhibitor binding site. Inhibitors PMP (yellow), CP (cyan) and GYKI (magenta) as well as side chains of residues mutated to alanines are shown as sticks. The pore axis is illustrated by a blue cylinder. (**D**,**E**) Representative whole-cell currents recorded at −60 mV membrane potential from HEK293 cells expressing wild type GluA2 (**D**) and mutant GluA2-S615A (**E**) receptors in response to 2 ms (blue) or 500 ms/1s (black) applications of 3 mM Glu alone or application of Glu in the continuous presence of 30 µM CTZ (green). The insets show normalized currents in response to 2 ms and 500 ms/1s applications of Glu alone fitted using single exponentials (red curves). (**F–H**) The average values of fractions of non-desensitized channels, *I*_SS_/*I*_Max_ and *I*_SS_/*I*_Peak_ (**F**) and time constants of deactivation, *τ*_Deact_ (**G**) and desensitization, *τ*_Des_ (**H**) for wild type and alanine-substituted GluA2 receptors. Error bars represent SEMs.

To probe the role of the ion channel extracellular collar in AMPA receptor gating, we first made alanine substitutions of residues in this region that contribute to or are adjacent to the noncompetitive inhibitor binding site (Fig. 1A–C). We expressed wild type and alanine-substituted GluA2i (flip) AMPA receptors in HEK293 cells and used whole-cell patch-clamp with fast solution exchange to record glutamate-activated currents. At −60 mV, 2-ms glutamate (Glu) application elicited a typical response of wild type GluA2 receptors: a rapidly activated inward current that quickly (*τ*_Deact_ = 1.76 ± 0.24 ms, n = 9) decayed to zero, mainly as a result of receptor deactivation (Fig. 1D, blue trace). Prolonged, 500-ms Glu application elicited an inward current that decayed in the continuous presence of glutamate more slowly (*τ*_Des_ = 7.15 ± 0.27 ms, n = 23), apparently due to receptor desensitization (Fig. 1D, black trace). Desensitization was completely blocked when Glu was applied in the presence of cyclothiazide (CTZ), a well-known positive allosteric modulator of AMPA receptors (Fig. 1D, green trace). To estimate the fraction of non-desensitized channels, we measured the ratio of the steady state current in the continuous presence of Glu (*I*_SS_) and the maximal current amplitudes in the presence (*I*_Max_) or absence (*I*_Peak_) of CTZ. For wild type GluA2 both ratios (*I*_SS_/*I*_Max_ = 0.028 ± 0.004, n = 17; *I*_SS_/*I*_Peak_ = 0.054 ± 0.005, n = 23) were comparable to previously reported values^2^.

Of 11 alanine mutants tested, F517A, which forms the “bottom” of the noncompetitive inhibitor binding site, showed the smallest fraction of non-desensitized channels (*I*_SS_/*I*_Max_ = 0.007 ± 0.002, n = 8; *I*_SS_/*I*_Peak_ = 0.013 ± 0.002, n = 9; that are approximately 4 times smaller than for the wild type receptors) and fastest rates of deactivation (*τ*_Deact_ = 1.17 ± 0.08 ms, n = 6, which is 1.5 times faster than for the wild type receptors) and desensitization (*τ*_Des_ = 5.03 ± 0.09 ms, n = 8, which is 1.42 times faster than for wild type receptors). On the other hand, the strongest effects on the measured parameters were observed for S615A in M3 (Fig. 1E). Compared to wild type, the S615A receptors had 27 times larger fraction of non-desensitized channels (*I*_SS_/*I*_Max_ = 0.719 ± 0.149, n = 4) and 5.7 times slower rates of deactivation (*τ*_Deact_ = 10.1 ± 1.9 ms, n = 5) and desensitization (*τ*_Des_ = 40.4 ± 3.4 ms, n = 6). Overall, the S615A mutant demonstrated slower kinetics and weaker desensitization than wild type AMPA receptors, reminiscent of NMDA receptors. Serine S615, which forms a hydrogen bond with the bound noncompetitive inhibitor^47^, is also the first residue in the highly conserved SYTANLAAF motif that includes the Lurcher site (the eighth residue, alanine)^34^ and has been previously reported to play an important role in iGluR gating^31^. Despite the side chain of S615 facing away from the ion channel pore, its alanine substitution has a profound effect on GluA2 gating. We tested alternative substitutions of this serine with cysteine (S615C) or tyrosine (S615Y), which showed similar but slightly weaker effects on gating parameters, compared to S615A (Suppl. Table 1). This result is very peculiar given that S615 is located in the tight environment of the helical bundle interface, and the side chain of alanine is smaller, while tyrosine is larger and cysteine is approximately the same size as serine.

Other alanine mutants showed intermediate effects between F517A and S615A (Fig. 1F–H, Suppl. Table 1). The second strongest effects after S615A compared to wild type receptors were observed for P520A. P520 is located at the elbow turn connecting the pre-M1 helix, oriented parallel to the membrane, and the M1 helix, oriented perpendicularly. Substitutions of P520 with residues other than alanine showed very small (P520G) or no currents (e.g., P520E, P520R and P520Y), supporting indispensable structural role of P520. Similar to P520A, P520G produced strong changes in the three measured gating parameters (Suppl. Table 1). Strong effects of P520A and P520G substitutions on gating suggest that P520 not only plays an indispensable structural role, but is also absolutely critical for iGluR gating. Supporting this conclusion, P520 is highly conserved (93%) among 1047 genes representing iGluR subunits^36^.

Importantly, S516A in pre-M1 and N791A in M4, two alanine substitutions that strongly affected PMP and GYKI binding to GluA2^47^ resulted in relatively small, but statistically significant differences in the fraction of non-desensitized channels and the time constants of deactivation and desensitization compared to wild type receptors (Fig. 1F–H, Suppl. Table 1). In order to explore the contribution of S516 and N791 to iGluR gating in more detail, we substituted them with different residues.

**Figure 2:**
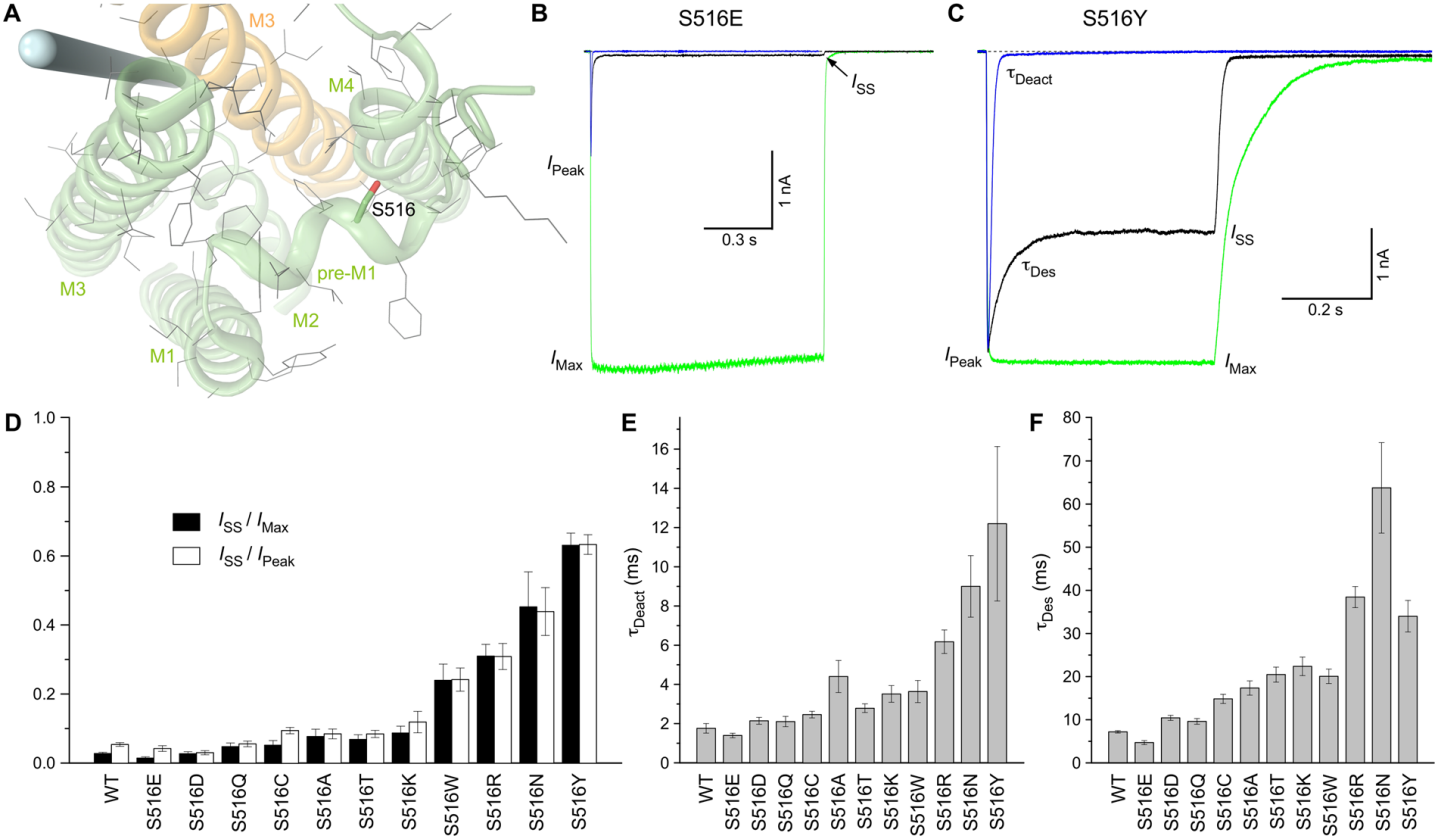
Substitutions of serine S516 with different amino acids. (**A**) Close-up view of the region encompassing noncompetitive inhibitor binding site, with S516 shown as thick (green and red) and other residues thin (grey) sticks. The pore axis is illustrated by a blue cylinder. (**B**,**C**) Representative superpositions of whole-cell currents recorded at −60 mV membrane potential from HEK293 cells expressing GluA2-S516E (**B**) and GluA2-S516Y (**C**) in response to 2 ms (blue) or 1s/500 ms (black) applications of 3 mM Glu alone or application of Glu in the continuous presence of 30 µM CTZ (green). (**D–F**) The average values of fractions of non-desensitized channels, *I*_SS_/*I*_Max_ and *I*_SS_/*I*_Peak_ (**D**) and time constants of deactivation, *τ*_Deact_ (**E**) and desensitization, *τ*_Des_ (**F**) for wild type GluA2 and mutant receptors. Error bars represent SEMs.

For S516, which is sticking up from the pre-M1 helix and facing M4 (Fig. 2A), we made 11 different substitutions and observed a broad range of effects on the fraction of non-desensitized channels and the rates of deactivation and desensitization. Indeed, compared to wild type receptors, the *I*_SS_/*I*_Max_ value varied from being 1.9 times smaller for S516E (Fig. 2B) to being 23 times larger for S516Y (Fig. 2C). Similarly, the rates of deactivation and desensitization were 1.3 and 1.5 times faster for S516E and 7 and 5 times slower for S516Y. Overall, the 11 different substitutions of serine S516 in pre-M1 showed a broad distribution of effects on gating parameters, with the strongest effects comparable to or exceeding those for the S615A substitution in M3 (Fig. 1F–H). In general, substitutions of S516 with negatively charged residues (Glu and Asp) demonstrated smaller fractions of non-desensitized channels and faster rates of deactivation and desensitization, while substitutions with positively charged and bulkier residues (Lys, Arg, Trp and Tyr) resulted in larger fractions of non-desensitized channels and slower rates of deactivation and desensitization (Fig. 2D–F, Suppl. Table 1). Nonetheless, the strong effects observed for S516N (e.g., 9 times slower desensitization than in wild type receptors) versus the relatively weak effects observed for S516Q and S516K argue that neither bulkiness nor charge of the side chain can alone explain the observed functional effects. Rather, interactions with surrounding residues in the local environment, which undergoes gating-related conformational changes, are the likely reasons for the observed pattern.

**Figure 3:**
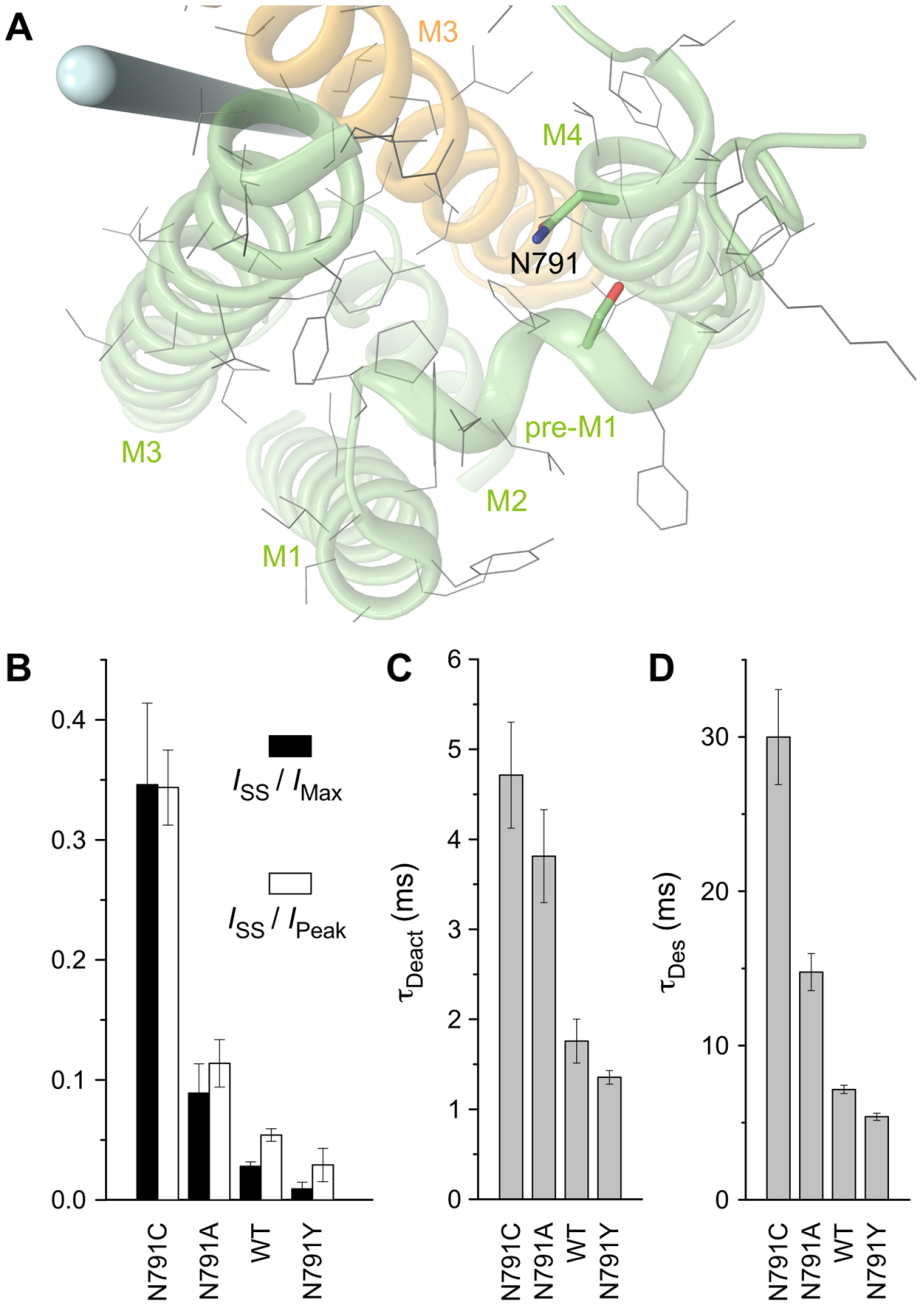
Substitutions of asparagine N791. (**A**) Close-up view of the region encompassing noncompetitive inhibitor binding site, with N791 shown as thick (green, blue and red) and other residues thin (grey) sticks. The pore axis is illustrated by a blue cylinder. (**B–D**) The average values of fractions of non-desensitized channels, *I*_SS_/*I*_Max_ and *I*_SS_/*I*_Peak_ (**B**) and time constants of deactivation, *τ*_Deact_ (**C**) and desensitization, *τ*_Des_ (**D**) for wild type GluA2 and mutant receptors. Error bars represent SEMs.

In addition to alanine, we made tyrosine and cysteine substitutions of N791, a residue sticking out of the top of the M4 helix and facing pre-M1 (Fig. 3A). For the tyrosine substitution, we were hoping to observe strong effects on gating parameters, similarly to S516Y. In fact, we did see significant changes in the parameter values but they were opposite of what we expected. Indeed, the N791Y mutation resulted in a smaller fraction of non-desensitized channels and faster rates of deactivation and desensitization compared to wild type and N791A receptors (Fig. 3B–D, Suppl. Table 1). However, the cysteine substitution N791C dramatically increased the fraction of non-desensitized channels and slowed down the rates of deactivation and desensitization. Apparently, substitutions with identical residues (alanine, cysteine and tyrosine) at S516 and N791, both located on the opposite sides of the same pre-M1-M4 interface, resulted in quite different effects on GluA2 gating (cf. Figs 2D–F and 3B–D).

**Figure 4:**
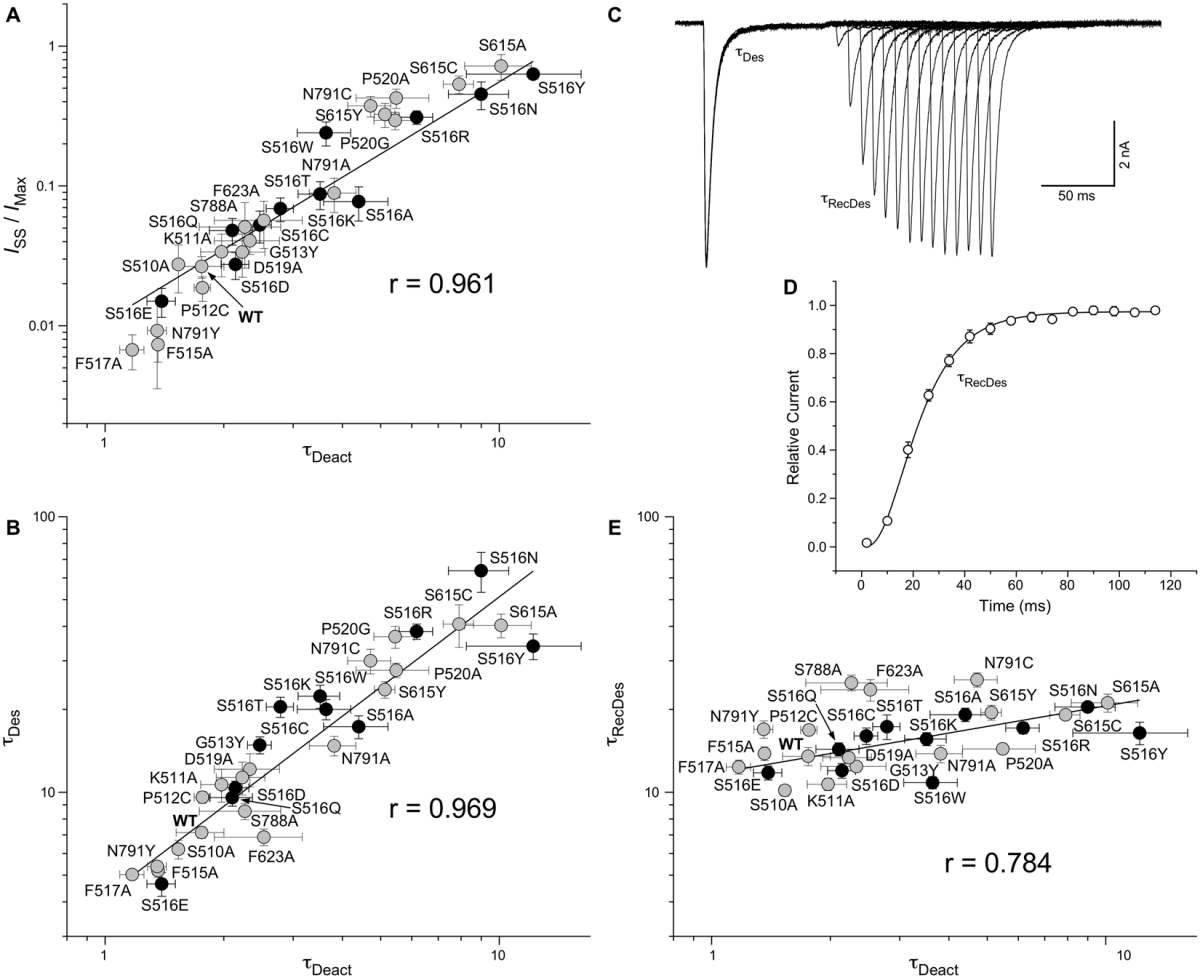
Correlations between gating parameters. (**A**,**B**,**E**) The fraction of non-desensitized channels, *I*_SS_/*I*_Max_ (**A**), time constant of desensitization, *τ*_Des_ (**B**), and time constant of recovery from desensitization, *τ*_RecDes_ (**E**), plotted against the time constant of deactivation, *τ*_Deact_. Each circle represents a mutant or wild type GluA2 receptor and labelled correspondingly. Circles representing S516 substituted mutants are black, while the other mutants and wild type receptors grey. Straight lines are linear fits for the entire pool of receptors with the Pearson’s correlation coefficient, r, shown on each plot. (**C**) Currents recorded from a cell expressing GluA2-F517A using a two-pulse protocol, in which an initial application of 3 mM Glu was made to produce steady-state desensitization and was repeated after allowing the channels to recover from desensitization for different length of time. The envelope of the peak currents evoked by the series of the second applications gives the time course of recovery from desensitization. (**D**) Mean recovery from desensitization measured for GluA2-F517A using the protocol illustrated in (**C**). The curve through the points is a fit with the Hodgkin-Huxley equation (see Methods) and the values of parameters *I*_max_ = 0.974 ± 0.005, *τ*_RecDes_ = 12.4 ± 0.7 ms and *m* = 3.55 ± 0.38 (*n* = 5). Error bars represent SEMs.

The broad range of changes in gating parameters caused by mutations in the ion channel extracellular collar illustrated in Figs 1–3 suggests that iGluR gating involves conformational rearrangements of the collar-contributing pre-M1, M3 and M4 segments. While it is difficult to explain the effects of particular mutations on gating without detailed understanding of conformational dynamics, one can notice that the three measured parameters, the fraction of non-desensitized channels and the time constants of deactivation and desensitization shown in Figs 1F–H, 2D–F and 3B–D, behaved somewhat similarly when comparing their values for different substitutions. To demonstrate this similarity, we plotted the values of *I*_SS_/*I*_Max_ (Fig. 4A) or *τ*_Des_ (Fig. 4B) against *τ*_Deact_. Indeed, strong correlations were observed for the entire pool of mutants (Pearson’s r = 0.961 for *I*_SS_/*I*_Max_ versus *τ*_Deact_ and r = 0.969 for *τ*_Des_ versus *τ*_Deact_) as well as separately for S516 mutants (0.970 and 0.936) or all except mutants of S516 (0.983 and 0.973). For each of the mutants, we also recorded the recovery from desensitization using a double-pulse protocol (Fig. 4C), fitted data using Hodgkin-Huxley equation (Fig. 4D) and defined the time constant of recovery from desensitization, *τ*_RecDes_ (Suppl. Table 1). Strikingly, the maximum (25.0 ± 1.6 ms for S788A) and minimum (10.2 ± 0.5 ms for S510A) values of *τ*_RecDes_ across all the substitutions were only 2.5-fold different. This difference was in stark contrast to 103-, 14- and 10-fold differences between the extreme values of *I*_SS_/*I*_Max_, *τ*_Des_ and *τ*_Deact_, respectively (Suppl. Table 1). Accordingly, correlation between *τ*_RecDes_ and *τ*_Deact_ (Fig. 4E) was much weaker, whether it was measured for the entire set of substitutions (Pearson’s r = 0.784), for S516 substitutions only (0.840) or for all but S516 substitutions (0.652).

To gain insight into possible conformational rearrangements at the ion channel extracellular collar, we employed MD simulations of the TMD domain in lipid membrane and water (Suppl. Fig. 1A). Because of the complexity and large size of the receptor, and because our goal was to characterize gating rearrangements in the TMD, only the TMD and linkers connecting it to the LBD were included in the model (Suppl. Fig. 1A). To facilitate opening of the ion channel pore, we introduced mutations in the M3 helix that either increased the ion channel open probability or weakened desensitization (see Methods), including the P520G mutation identified in the present study, which was reversed at the late stages of the simulation. Briefly, we performed initial equilibration MD simulations of the apo state closed channel structure (5L1B)^47^ in a POPC lipid bilayer and water, then the subsequent equilibrium MD simulations of the wild type and mutant structures in the absence of all restraints (Suppl. Fig. 1B). The equilibrated closed channel structure was then used in targeted MD simulations to produce an open state channel (Suppl. Fig. 1C,D). To promote pore opening, we used a minimally invasive targeted MD procedure that included four-fold symmetrical application of biasing potentials to the channel constriction region formed by the M3 helix bundle, designed to increase the distance between the adjacent M3 helixes, and hence, the channel radius in the area of the constriction (see Methods, Suppl. Fig. 1). This procedure implied that the driving force for channel opening is an outward motion of the pore-forming M3 helices. While such an assumption is generally accepted in the field of tetrameric ion channels, there may be other alternatives to the open-channel model.

**Figure 5:**
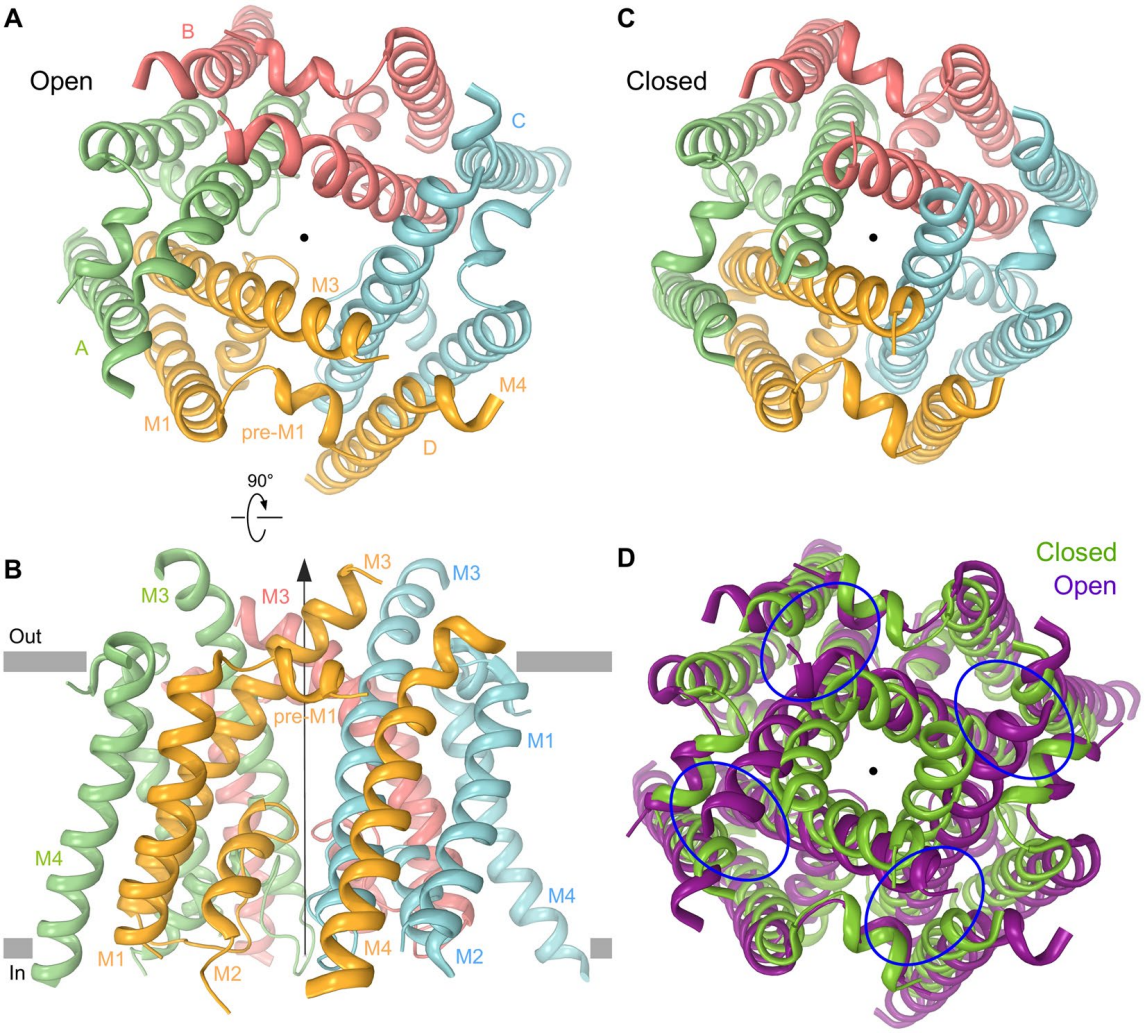
MD simulated ion channel open state. (**A**,**B**) MD simulated structure of GluA2 ion channel in the open state (see Methods and text) viewed extracellularly (**A**) or parallel to the membrane (**B**). The pore axis is illustrated by a black arrow. (**C**) Closed channel from GluA2_apo_ structure (PDB ID: 5L1B) viewed extracellularly. (**D**) Superposition of the ion channel open (purple) and closed (green) conformations from (**A** and **B**) respectively. Blue ovals designate the noncompetitive inhibitor binding pockets.

The targeted MD simulations were performed until the pore became comfortably solvated and an uninterrupted water channel connected the two sides of the membrane. At the end of our simulations, the diameter of the pore was large enough to conduct potassium ions (Suppl. Fig. 2). We tested the ability of the open pore model to conduct potassium ions by increasing K^+^ concentration at the channel entrance and observing events of ion permeation directly in the equilibrium MD simulations (Suppl. Movies 1 and 2). Therefore, we believe that our final model (Fig. 5A,B) is a faithful representation of the GluA2 ion channel in the open state. Compared to the GluA2 apo state structure (closed channel, 5L1B)^47^ (Fig. 5C) the open state model lacks 4-fold symmetry of the ion channel extracellular portion and instead has a 2-fold symmetrical architecture. The extension of the 2-fold symmetry from the LBDs deeper into the TMD is expected based on the previous structural and functional work^26–28, 30, 49^ and originates from the longer M3 helices in diagonal subunits A and C moving farther away from the centre of the pore than the shorter M3 helices in subunits B and D (cf. Fig. 5A and C). The covariance and principal component analysis (PCA) of the TMD domain transformation during channel opening transition provides a more complete and detailed picture of the complicated 2-fold symmetrical relative movement of the M1, pre-M1, M3 and M4 segments (Suppl. Figs 3–5 and Suppl. Movies 3–6). Assuming similarity of the closed pore conformations in the apo and desensitized states, the predicted remodelling of noncompetitive inhibitor binding pockets (Fig. 5D) strongly supports the conclusions of our mutagenesis and functional experiments (Figs 1–4) that iGluR gating involves significant rearrangement of the contributing pre-M1, M3 and M4 segments at the ion channel extracellular collar.

**Figure 6:**
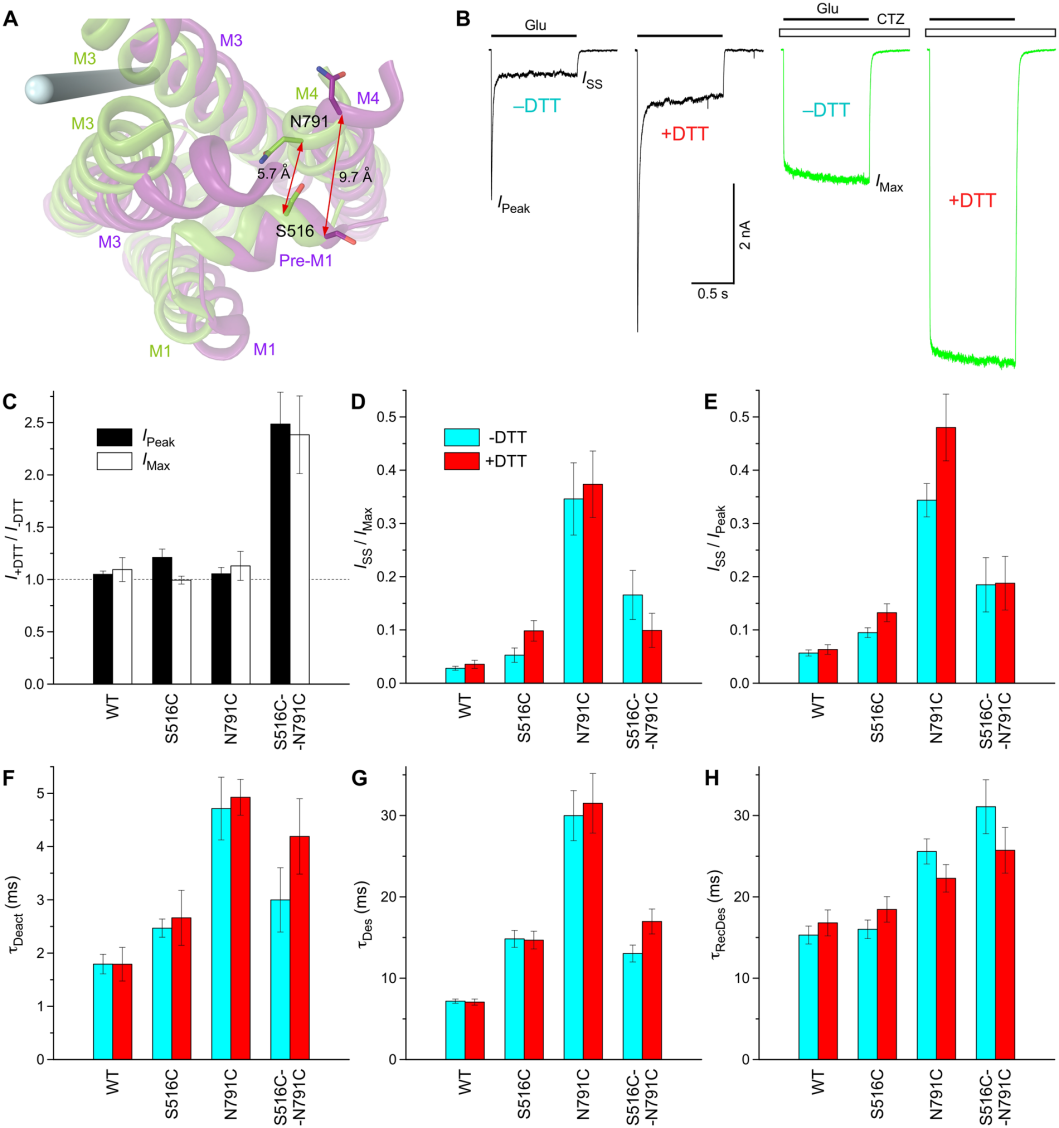
Crosslink between S516C and N791C. (**A**) Close-up view of the region encompassing noncompetitive inhibitor binding site in superposed structures of the MD simulated open (purple) and GluA2_apo_ closed (PDB ID: 5L1B) states. Side chains of S516 and N791 are shown as sticks. The pore axis is illustrated by a blue cylinder. Distances between C_α_’s of S516 and N791 in the two structures are indicated by red arrows and labelled. (**B**) Representative whole-cell currents recorded at −60 mV membrane potential from HEK293 cells expressing GluA2-S516C-N791C receptors in response to 1s applications of 3 mM Glu alone (black) or application of Glu in the continuous presence of 30 µM CTZ (green), in non-reducing (−DTT) or reducing (+1 mM DTT) conditions. (**C**) The ratio of the maximal current amplitudes measured in reducing versus non-reducing conditions in the absence (*I*_Peak_, filled bars) or presence (*I*_Max_, open bars) of CTZ for wild type (WT), single cysteine (S516C and N791C) or double cysteine (S516C-N791C) substituted GluA2 receptors. (**D–H**) The average fractions of non-desensitized channels, *I*_SS_/*I*_Max_ (**D**) and *I*_SS_/*I*_Peak_ (**E**), and the time constants of deactivation, *τ*_Deact_ (**F**), desensitization, *τ*_Des_ (**G**), and recovery from desensitization, *τ*_RecDes_ (**H**) measured in non-reducing (−DTT, cyan bars) or reducing (+DTT, red bars) conditions. Error bars represent SEMs.

We next used cysteine crosslinking to probe the conformational changes predicted by our MD simulated open state model. We noticed an increase in the distance between C_α_’s of S516 and N791 from approximately 6 Å in the GluA2 apo state structure to 9–12 Å in the predicted open state structure (Fig. 6A). Given strong effects of the S516 and N791 substitutions on GluA2 gating (Figs 2 and 3), we tested whether ion channel opening is altered by crosslinking of the corresponding substituted cysteines.

In fact, the double cysteine substituted S516C-N791C receptors demonstrated nearly 2.5-fold increase in the current amplitude in reducing compared to non-reducing conditions (Fig. 6B). In contrast, wild type and single cysteine substituted S516C or N791C receptors did not show significant differences (Fig. 6C). While the values of many gating parameters were different between the wild type, S516C, N791C and S516C-N791C receptors, these differences were not significant between reducing and non-reducing conditions (Fig. 6D–H). The apparent inhibition of ion channel opening by the S516C-N791C crosslink (Fig. 6B) might therefore result from slowing down of the rate of channel opening. While detailed understanding of the mechanism of the closed state stabilization by the S516C-N791C crosslink requires additional studies, the crosslinking experiments strongly support our MD simulated open state model that predicts separation of the pre-M1 and M4 segments at the ion channel extracellular collar upon channel opening.

## Discussion

The results of our experiments show that mutations at the extracellular collar of the iGluR ion channel produce strong effects on gating and that the corresponding changes in gating parameters have a distinct pattern, specific to the collar region. For example, strikingly different behaviour was observed for mutations made in the LBD, where the deactivation and recovery from desensitization rates were strongly correlated, while no correlation was observed between the rates of desensitization and recovery from it^50–52^. Therefore, for the LBD mutants, it is likely that the same molecular interactions slowed recovery from desensitization and ion channel closure. In contrast, mutations in the ion channel extracellular collar described in this study showed strong correlation between the fraction of non-desensitized channels and the deactivation and desensitization time constants (Fig. 4A,B). Given the adjacent location of the collar to the ion channel pore, such correlation intuitively has sense since any changes in the protein structure here that promote channel closure would work similarly for deactivation and desensitization, both leading to current reduction. On the other hand, these parameters correlated weakly with the recovery from desensitization time constant, which did not vary strongly between mutants (Fig. 4E). This suggests that recovery from desensitization takes different pathway of conformational rearrangements. Overall, the distinct gating patterns observed for mutations in the LBD and ion channel extracellular collar emphasize that these domains act via different allosteric mechanisms to regulate AMPA receptor function.

Since there was no clear dependence of changes in gating parameters on the type of side chain substitution, we conclude that the effect of each individual substitution depends on (1) location of the substituted residue, (2) interaction with the local environment and (3) changes in this local environment during gating. In the context of the available apo state crystal structure of GluA2 (Fig. 1A–C), the first two factors do not provide clear explanation for the observed effects. Perhaps the third factor is the most important for understanding the strong effects of mutations at the extracellular collar of the ion channel on GluA2 gating. Indeed, the ion channel extracellular collar has a perfect location for the most efficient allosteric regulation: it is not directly involved in the permeation pathway but surrounds it right at the gate region formed by the M3 helices bundle crossing. Opening of the ion channel requires the bundle to move apart to open the pore for passage of permeant ions. In turn, movement of the M3 helices will change their relative positioning with respect to the surrounding segments of the collar region – pre-M1, M1 and M4. Since the residues mutated in our study are located at the interfaces between pre-M1, M3 and M4, their environment is expected to undergo drastic changes during gating. The MD simulation experiments strongly support this conclusion.

Our MD simulated open state model (Fig. 5A,B) demonstrates significant structural rearrangements at the ion channel extracellular collar relative to the apo state structure (Fig. 5C) that cause profound transformation of the noncompetitive inhibitor binding pockets (Fig. 5D, Suppl. Movies 3–6). Consistent with previous mutagenesis studies^49^, the open state model has 2-fold (Fig. 5A) rather than 4-fold rotational symmetry (Fig. 5C). Comparing the open state model to the apo state structure, M4 is predicted to separate from the pre-M1 helix as the channel opens (Fig. 6A). The reduced hydrophobic interaction between F517 and V795 caused by the F517A mutation might be the reason for increased desensitization and higher rates of deactivation and desensitization observed for this mutant (Fig. 1). On the other hand, the distance between C_α_’s of S615 in one M3 segment and L624 in the neighbouring M3 segment decreases from 10–11 Å in the closed state to *~*6 Å for the A and C subunits in the predicted open state (for the B and D subunits, the distance stays the same, 10–11 Å). The substitution of S615 with alanine or tyrosine therefore might increase the hydrophobic interaction with L624, thus promoting stability of the open state and inhibiting desensitization (Suppl. Table 1). On the other hand, predicted separation of S516 and N791 upon channel opening (Fig. 6A) is also accompanied by E627 moving 1–2 Å closer to S516 and 5–7 Å closer to N791. Correspondingly, substitutions of S516 with bulky side chain residues promoting separation with N791 (S516Y or S516W) or positively charged residues capable of forming an electrostatic interaction with E627 (S516R or S516K) can promote channel opening or reduce desensitization (Fig. 2). Conversely, substitution of N791 with the bulky hydrophobic tyrosine versus the smaller alanine and cysteine might disfavour the open state by clashing with negatively charged E627 (Fig. 3). Interestingly, the previously identified E627R mutation that promoted AMPA receptor desensitization^35^ might destabilize the open state by either unfavourable interaction with the hydrophobic environment of the noncompetitive antagonist binding pocket or through electrostatic repulsion with K511. Similarly, substitution of L521 in the “hydrophobic box” with a more hydrophobic phenylalanine^36^ can promote closer proximity of M1 and M3, thus stabilizing the open and destabilizing the desensitized states, respectively. Our predicted all-atom open state model of the TMD (Fig. 5) is therefore consistent with the results of the present and previous mutagenesis and functional studies. In the absence of high resolution crystallographic or cryo-EM structures of the AMPA receptor TMD in the open state, our predicted model can be instrumental for iGluR structure-function analysis, assessing the energetics of the conformational transitions in the TMD, as well as for predicting gating rearrangements in the context of the full-length receptor.

In summary, we observed a distinct pattern of effects produced by single-residue substitutions at the ion channel extracellular collar on gating parameters that suggests a unique and specific role of this region in AMPA receptor gating. Using MD simulations, we constructed a structural model of the GluA2 open state that is consistent with the present and previous mutagenesis studies, and can be employed in future structure-function analysis. We conclude that the ion channel extracellular collar represents a hub for powerful allosteric modulation of AMPA receptor function that can be used as a target for developing novel therapeutics.

## Methods

### Constructs and expression

The full length rat GluA2i (flip) (NP_058957) subunit (also known as GluRBi or GluR2i)^53, 54^, including the native signal peptide, was introduced into a plasmid for expression in eukaryotic cells^54^ that was engineered to produce green fluorescent protein via a downstream internal ribosome entry site^27^. Point mutations were made using conventional mutagenesis techniques and mutant receptor DNA was sequences over the entire length of the GluA2 coding region. Human embryonic kidney HEK293 cells grown on glass cover slips in 35-mm dishes were transiently transfected with 1–5 μg of plasmid DNA using Lipofectamine 2000 Reagent (Invitrogen).

### Electrophysiology

Recordings were made 24 to 96 hours after transfection at room temperature. Currents from whole cells, typically held at a −60 mV potential, were recorded using Axopatch 200B amplifier (Molecular Devices, LLC), filtered at 5 kHz and digitized at 10 kHz using low-noise data acquisition system Digidata 1440 A and pCLAMP software (Molecular Devices, LLC). The external solution contained (in mM): 140 NaCl, 2.4 KCl, 4 CaCl_2_, 4 MgCl_2_, 10 HEPES pH 7.4 and 10 glucose; 7 mM NaCl was added to the extracellular activating solution containing 3 mM L-glutamate (Glu). The internal solution contained (in mM): 150 CsF, 10 NaCl, 10 EGTA, 20 HEPES pH 7.3. Rapid solution exchange was achieved with a two-barrel theta glass pipette controlled by a piezoelectric translator. Typical 10–90% rise times were 200–300 µs, as measured from junction potentials at the open tip of the patch pipette after recordings. Since we recorded GluA2-mediated currents in the whole-cell mode, the time constants of deactivation and desensitization were somewhat larger than the corresponding values measured using outside-out recordings^35, 36, 50–52^. Nevertheless, despite the apparent overestimation of the absolute values of these parameters, we believe that their differences faithfully reflected differences in kinetics of GluA2 mutants. Similarly, the maximal current amplitude measured in the absence of CTZ (*I*_Peak_) might be underestimated. Thus, to more reliably determine the fraction of non-desensitized channels, we measured not only the *I*_SS_/*I*_Peak_ ratio but also the *I*_SS_/*I*_Max_ ratio, where both *I*_SS_ and *I*_Max_ current amplitudes were independent of the speed of solution exchange. Data analysis was performed using the computer program Origin 9.1.0 (OriginLab Corp.). Recovery from desensitization recorded in two-pulse protocols was fitted with the Hodgkin-Huxley equation^55^:

$I={({{I}_{max}}^{1/m}-({{I}_{max}}^{1/m}-{{I}_{0}}^{1/m})\times \exp (-t/\tau ))}^{m},$ where *I* is the peak current at a given interpulse interval, *t*, *I*_max_ is the peak current at long interpulse intervals, *I*_0_ is the current at zero time, *τ* is the recovery time constant and *m* is an index that corresponds to the number of kinetically equivalent rate-determining transitions that contribute to the recovery time course.

### MD simulations

All simulations were carried out using the molecular dynamics software package Amber12^56^ using the pmemd.cuda program and the ff12SB force field^57^. We performed the MD simulations of the GluA2 TMD with linkers to LBD domain in POPC membrane^58^ and water (TIP3P). All simulations were performed with 2-fs integration time step, pressure and temperature were maintained at 1 bar and 300 K respectively. We used the Langevin thermostat with damping coefficient of 1 ps^−1^ and anisotropic pressure scaling with pressure relaxation time of 1 ps. For all simulations, we employed periodic boundary conditions and all-atom wrapping. For the Lennard-Jones and Coulombic interactions, Particle Mesh Ewald method was used as implemented in Amber with the cut off distance of 10 Å.

The crystal structure of GluA2 apo state (PDB ID: 5L1B)^47^ was used as a starting conformation for all MD simulations. The full-length protein was truncated to include the TMD and LBD-TMD linkers only, namely residues 509–558, 567–635 and 786–817 (numbering is the same as for the GluA2_Del_ construct^47^). All atoms missing in the crystal structure were modelled using tleap program of Amber12 package. The protein was embedded in an equilibrated bilayer membrane consisting of 240 1-palmitoyl-2-oleoyl-sn-glycero-3-phosphocholine (POPC) lipids using the CHARMM-GUI membrane builder tool^59^. Water molecules were added to the ion channel pore. The resulting system was solvated by bulk water. K^+^ ions were added randomly within the solvent to neutralize the total charge of the system. The resulting simulation system consisted of 560 residues of the GluA2 tetramer (TMD and LBD-TMD linkers), 240 POPC lipid molecules, 7081 TIP3P water molecules and 12 K^+^ counter ions. For initial equilibration, the energy of the system was slightly minimized (7500 steps), then the system was heated to 300 K and an equilibration simulation was run for 20 ns. During equilibration, all protein C_α_ atoms were restrained using harmonic potential at their initial positions. The force constant to restrain C_α_ atoms (*k*) was decreased gradually during equilibration simulation from 20 to 0.5 kcal mol^−1^ Å^−2^. After equilibration, an equilibrium MD simulation was performed in the absence of all constrains on the protein.

The equilibrated model was used as a starting point for development of an open channel model. First, we introduced mutations in the M3 helix that either increase ion channel open probability or weaken desensitization^31^: T617S, A618C, A621S and A622Y. In addition, P520G substitution was introduced based on the present study. The resulting system was further equilibrated. The energy of the system was slightly minimized to remove atom clashes (7500 steps) and then equilibrated in MD simulations for 25 ns. In particular, the system was first heated to 300 K and equilibrated for 10 ns, while all the C_α_ atoms were restrained at their initial positions with the force constant *k* = 0.5 kcal mol^−1^ Å^−2^. Thereafter, all restraints were removed, except the restraints on the TMD-LBD linkers, which were maintained for further 15 ns with *k* = 0.5 kcal mol^−1^ Å^−2^.

The equilibrated structure of the mutant TMD was used as a starting point for the targeted MD simulations. To initiate channel opening simulation in the absence of known end point (open state) structure, we used a targeted (steered) MD protocol to apply a harmonic biasing potentials to the top segments of the M3 helices to increase the radius of the pore. These segments included the pore lining portions SYTANLAAFLTVERMV of the longer M3 helices (subunits A and C) and SYTANLAAFLTV of the shorter M3 helices (subunits B and D). The distances between the centers of mass (COM) of the C_α_ atoms of the sequence fragments listed above were set to increase in a pairwise manner between each pair of the adjacent subunits: A-D, B-A, C-B and D-C. The initial target distance was set to increase the distance between the adjacent subunits by 2 Å with a harmonic force of 15 kcal mol^−1^ Å^−2^ over 30 ns. The hydrogen bonds and the dihedral angles of all TMD helices were then harmonically restrained for 10 ns with a force constant of 5 kcal mol^−1^ Å^−2^ to ensure retaining of the secondary structure. The system was then equilibrated for 100 ns, while maintaining the A-D, B-A, C-B and D-C inter-subunit distances with the force of 15 kcal mol^−1^ Å^−2^. The A-D, B-A, C-B and D-C inter-subunits distances were then set to increase by an additional 1 Å over 20 ns with the harmonic force of 15 kcal mol^−1^ Å^−2^. Thirteen potassium ions were then added in the vicinity of the M3 bundle crossing (the system was neutralized with 13 Cl^−^ ions) in order to facilitate the ion channel opening by exerting osmotic pressure on a nearly open channel. The resulting system was equilibrated for 200 ns while maintaining the A-D, B-A, C-B and D-C inter-subunit distances restrained with the force constant of 15 kcal mol^−1^ Å^−2^. The A-D, B-A, C-B and D-C inter-subunits distances were set to increase by an additional 1.5 Å over 20 ns and one potassium ion did permeate through the channel constriction indicating that the channel model became open for ion permeation (see Suppl. Movies 1 and 2). The resulting system was equilibrated for 30 ns while A-D, B-A, C-B and D-C inter-subunit distances restrained with the force constant of 15 kcal mol^−1^ Å^−2^. The A-D, B-A, C-B and D-C inter-subunits distances were then set to increase by an additional 2.5 Å over 90 ns with the harmonic force of 15 kcal mol^−1^ Å^−2^. Next, the P520G mutation was reversed to return to the original proline and the system was equilibrated for an additional 50 ns while restraining A-D, B-A, C-B and D-C inter-subunit distances with a weak force constant of 2 kcal mol^−1^ Å^−2^. During the last 50 ns of the simulations, the channel remained in the open conformation and did not further change its conformation indicating good convergence of the simulations (Suppl. Fig. 1C). In summary, the all atom model of GluA2 was first equilibrated for 45 ns in the closed state and the subsequent modelling of the open state was performed for an additional time of 570 ns using targeted MD simulations.

## Electronic supplementary material

Supplementary Information

Supplementary Movie 1

Supplementary Movie 2

Supplementary Movie 3

Supplementary Movie 4

Supplementary Movie 5

Supplementary Movie 6
