# Supplementary material for: Role of the Ion Channel Extracellular Collar in AMPA Receptor Gating
Source: Sci Rep. 2017 Apr 21;7:1050. doi: 10.1038/s41598-017-01146-z (PMC5430913; doi:10.1038/s41598-017-01146-z)
Supplement: Supplementary file 1 — Supplementary Information [file 41598_2017_1146_MOESM1_ESM.pdf]

Supplementary Information

(Supplementary Figures 1-5, Supplementary Table 1 and Supplementary Movie Legends)

Role of the Ion Channel Extracellular Collar in AMPA Receptor Gating

Maria V. Yelshanskaya, Samaneh Mesbahi-Vasey, Maria G. Kurnikova and Alexander I. Sobolevsky

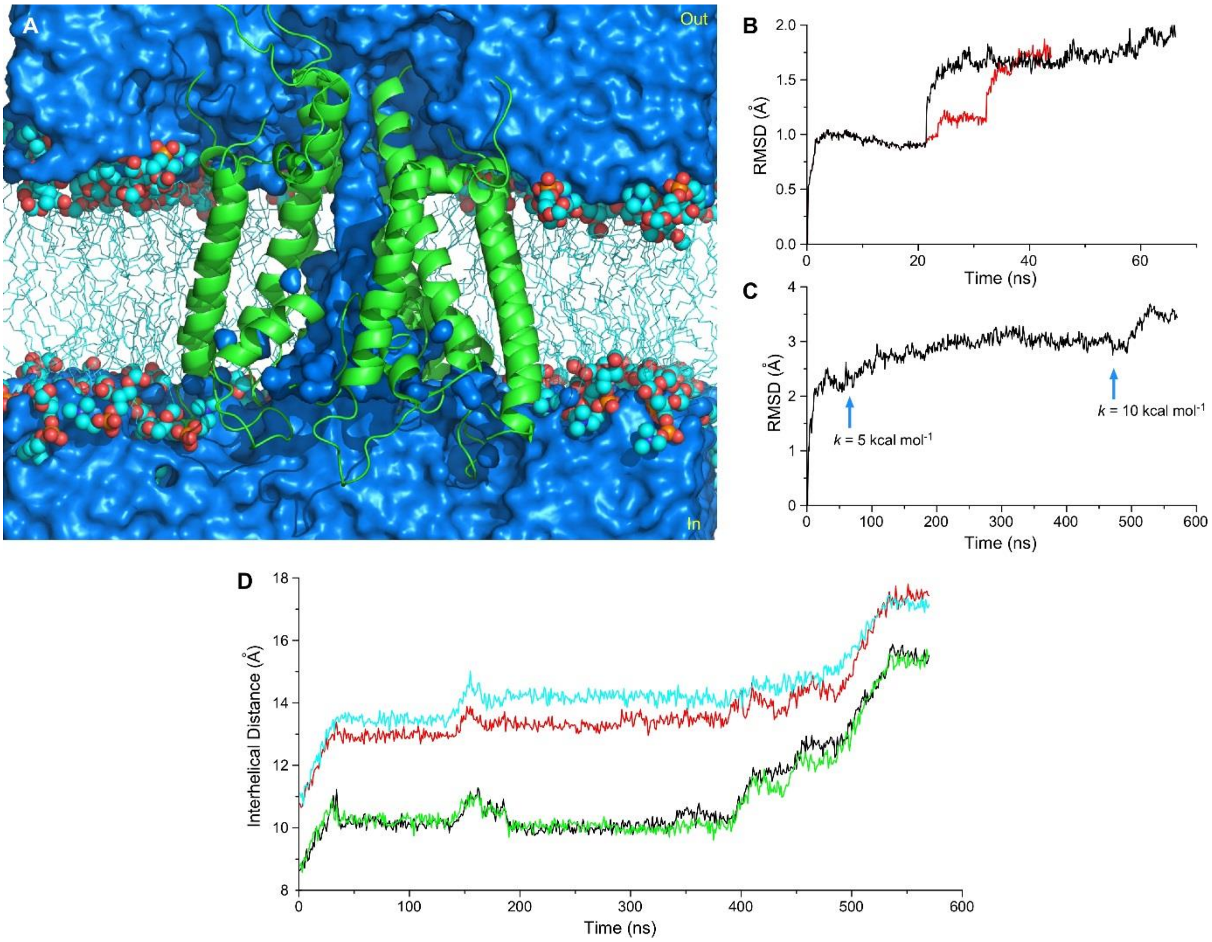

Supplementary Figure 1. Setup and progress of MD simulations. ***A***, Simulated AMPA receptor TMD in POPC lipid bilayer and water in the closed channel conformation. The protein is shown as cartoon (green), lipid molecule hydrophobic tails as sticks (cyan) and lipid head groups as spheres with carbon atoms colored cyan, oxygen red and phosphorus orange. Water is represented by blue continuous medium. Water and lipids are truncated in the page plane to expose the channel. The counter ions are not shown. For detailed description, see Methods. ***B***, Equilibration and equilibrium MD simulations of the closed-channel TMD. The root mean squared deviation (RMSD) calculated relative to the initial wild type (black line) or mutated (red line) structures as an average for all C_α atoms in the TMD helical core, including the segments M1, M3 and M4, is shown as a function of time. During the first 20 ns of equilibration, the harmonic constrains (see Methods) were used to preserve the structural conformation. After 20 ns, all constrains were removed. Both wild type and mutant structures were stable and preserved their closed-channel conformations. As seen in the graph, the relaxation of the structure is small, typical of a well folded protein. ***C***, Targeted MD simulation of the channel pore opening. Shown is the average RMSD for all C_α atoms in the TMD helical core calculated relative to the targeted MD starting structure (a structure taken at 21 ns of equilibrium simulation of the closed channel as shown in ***B***). Arrows indicate times when the force of the steering potential has been changed. Note, despite steering, the overall structure of the protein changes very little, an indicative of a “well-behaving” conformational transition with no noticeable distortion of the structure. ***D***, Inter-helical distances calculated for the centers of mass (COM) of the M3 segments (see Methods) that were pulled in pairwise fashion during targeted MD simulations of the channel opening. Shown are the distances between subunits A and B (red), B and C (green), C and D (blue) and D and A (black), all of which correspond to the application of the bias in targeted MD as described in Methods.

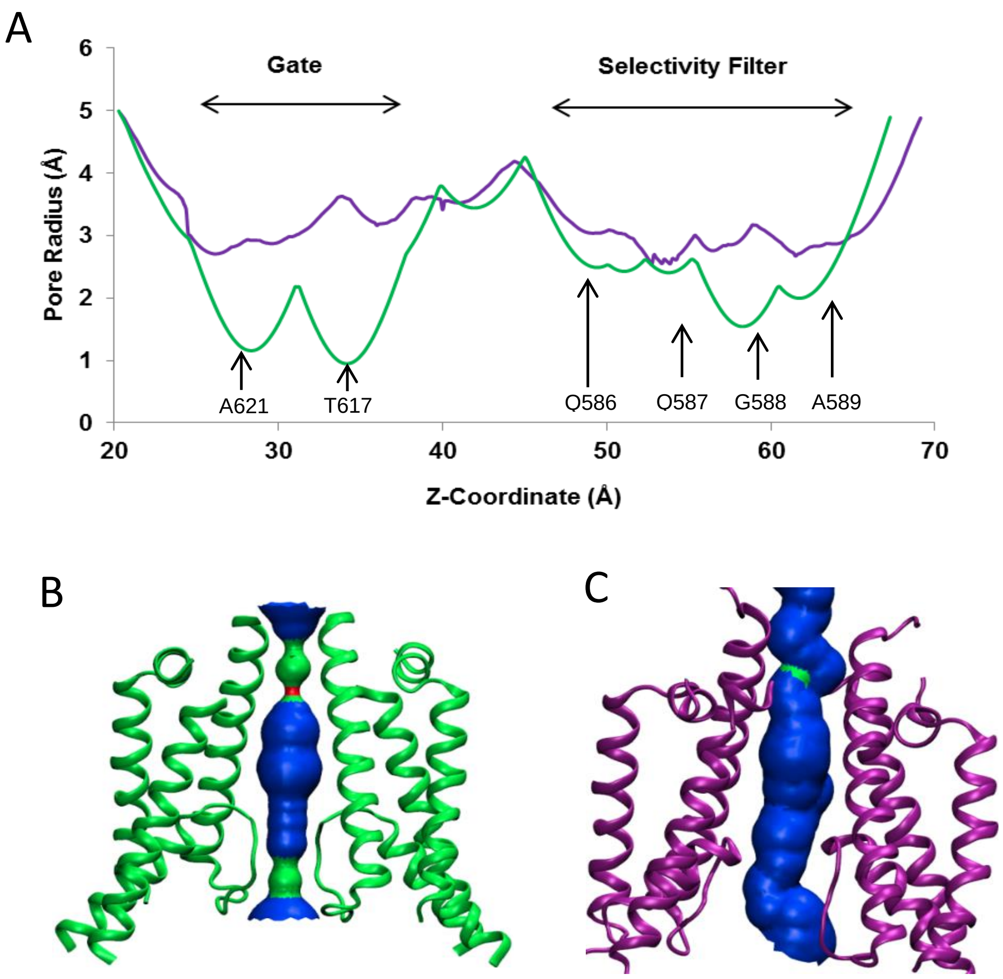

Supplementary Figure 2. Pore profiles for the ion channel closed and open states. ***A.*** The pore radius as a function of coordinate along the central pore axis (Z) calculated using HOLE[56] for the apo state crystal structure (PDB ID: 5L1B, *green*) and the MD simulated open state structure (*purple*). Residues that form pore constrictions are indicated. ***B-C*** Surface representation of the ion conduction pathway in the apo state structure (***B***) and the open state model (***C***) coloured according to the pore radius (red < 1 Å < green < 2.5 Å < blue). Two of four AMPA receptor subunits are shown in cartoon representation, with the front and back subunits removed for clarity.

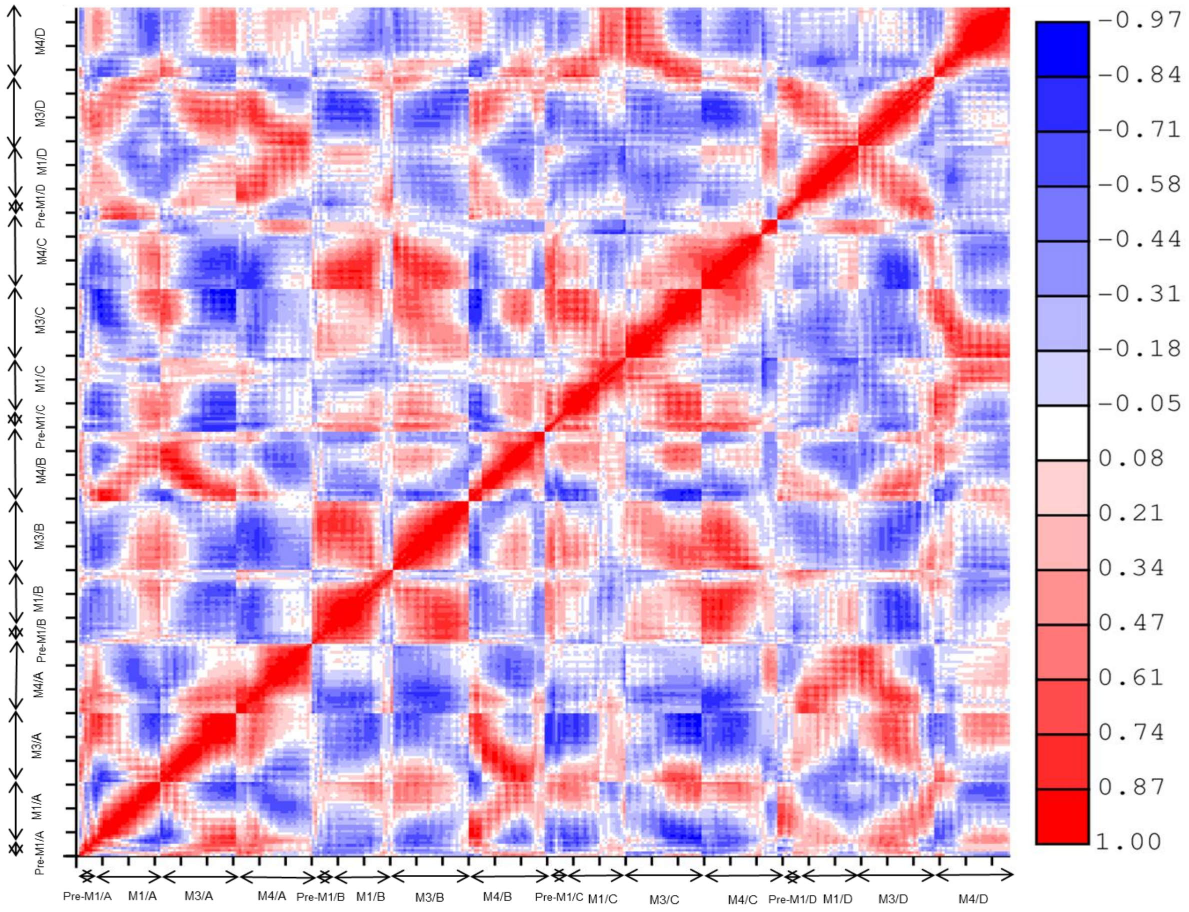

Supplementary Figure 3. Covariance matrix of the pairwise atomic fluctuations in targeted MD simulations. The matrix was calculated relative to the average structure along the opening trajectory. TMD helices and the corresponding subunits (A, B, C or D) are indicated with arrows. The axis scales are 10 residues between the tick marks. Correlated and anti-correlated motions are coloured red and blue, respectively.

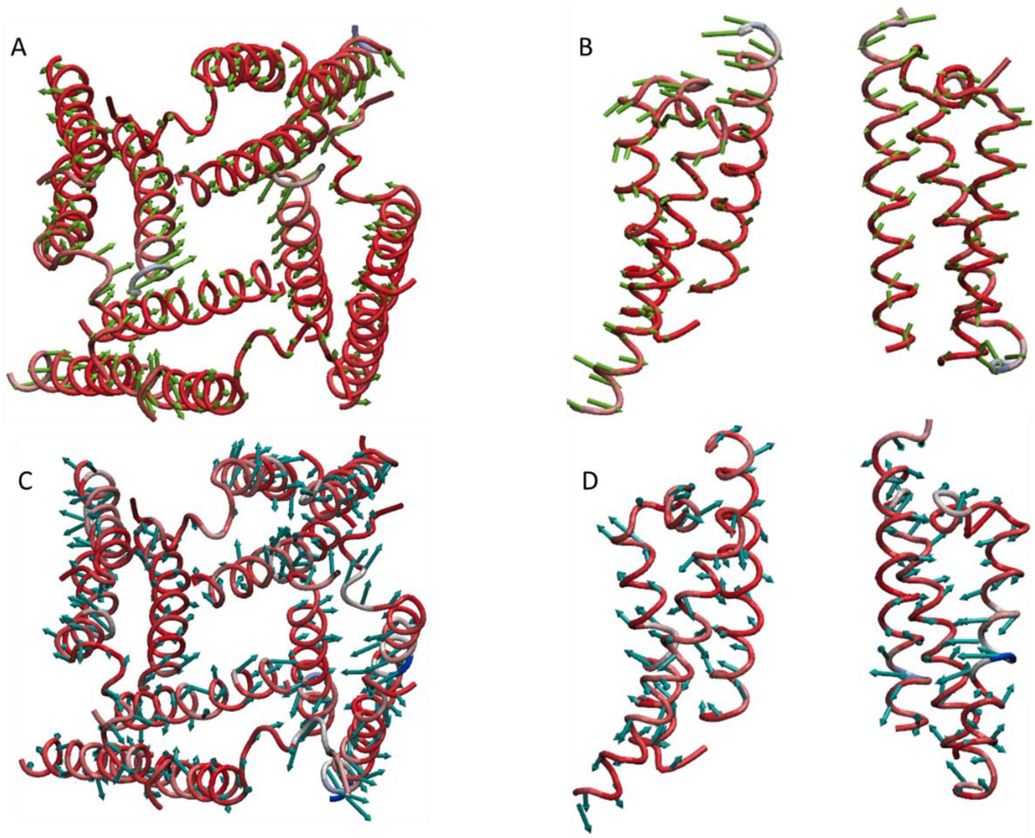

Supplementary Figure 4. Main principal component vectors. The principal component vectors (eigenvectors) PC1 (***A***-***B***) and PC2 (***C***-***D***) that correspond to the covariance matrix shown in Suppl. Fig. 3 are superposed on the average structure along the opening trajectory viewed extracellularly (***A*** and ***C***) or parallel to the membrane (***B*** and ***D***). In ***B*** and ***D***, only two of four AMPA receptor subunits are shown, with the front and back subunits removed for clarity.

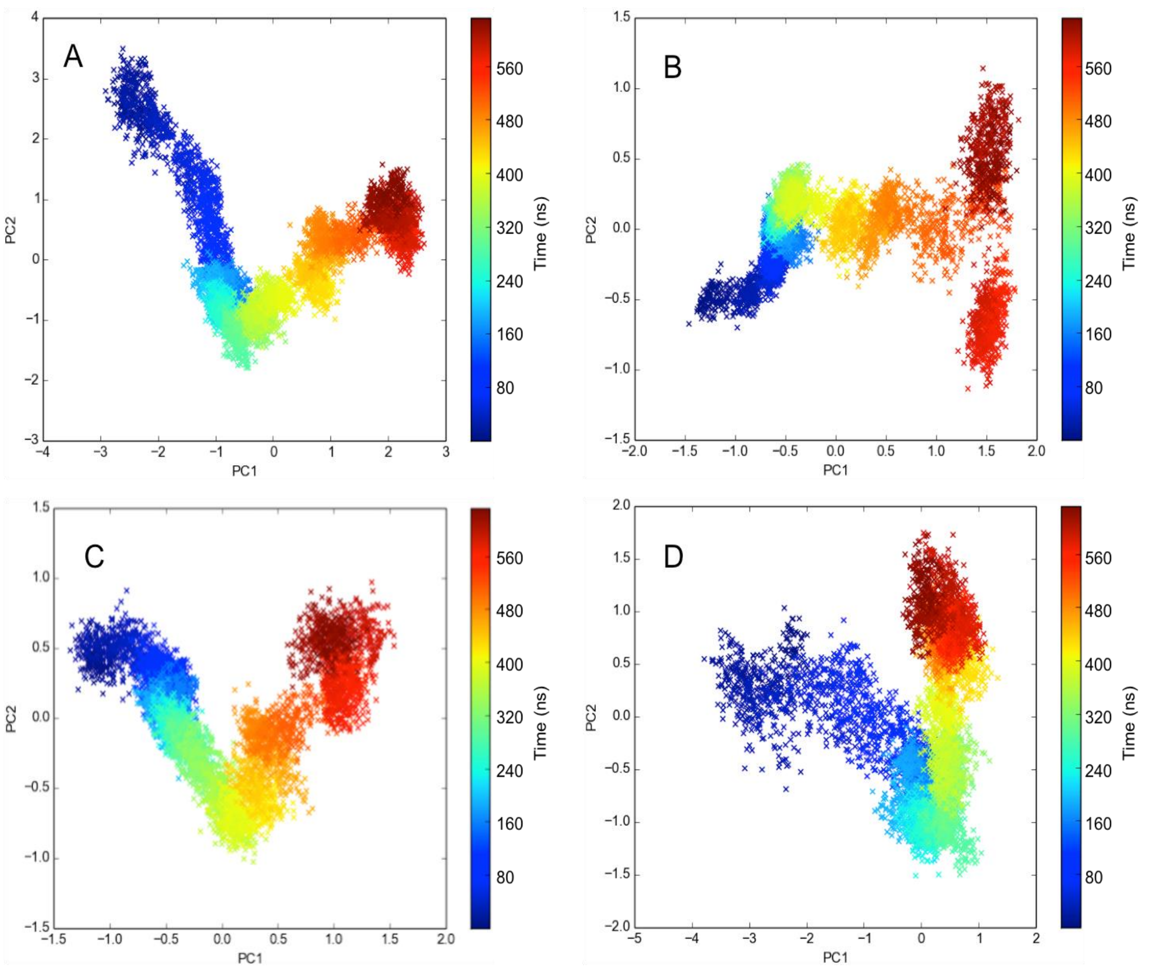

Supplementary Figure 5. Targeted MD trajectory projection onto the space of two main principal component vectors. The channel opening trajectory is projected onto two-dimensional space of the PC1 and PC2 principal component vectors that correspond to the covariance matrix of Suppl. Fig. 3 and are illustrated in Suppl. Fig. 4 and animated in Suppl. Movies 3-6. Colour indicates progression in time during the simulation. ***A***, Principal component analysis (PCA) for the motion of the M1, M3 and M4 helixes. Contributions to the variance of PC1 and PC2 are 32.60% and 17.85%, respectively. ***B***, PCA for the M3 helices. Contributions to the variance of PC1 and PC2 are 59.65% and 8.93%, respectively. ***C***, PCA for the M1 helices. Contributions to the variance of PC1 and PC2 are 32.84% and 12.32%, respectively. ***D***, PCA for the M4 helices. Contribution to the variance of PC1 and PC2 are 31.15% and 21.73%, respectively.

Supplementary Table 1. Electrophysiological parameters for wild type (WT) and mutant GluA2 receptors

Mutant	I_{SS} / I_{Max}	I_{SS} / I_{Peak}	τ_{Deact}, ms	τ_{Des}, ms	τ_{RecDes}, ms
WT	0.028 ± 0.004 (n=17)	0.054 ± 0.005 (n=23)	1.76 ± 0.24 (n=9)	7.15 ± 0.27 (n=23)	15.3 ± 1.1 (n=14)
S510A	0.027 ± 0.010 (n=5)	0.045 ± 0.011 (n=9)	1.53 ± 0.05 (n=8)	6.22 ± 0.48 (n=9)	10.2 ± 0.5 (n=6)
K511A	0.034 ± 0.011 (n=4)	0.058 ± 0.012 (n=6)	1.97 ± 0.23 (n=5)	10.7 ± 1.5 (n=6)	10.7 ± 0.6 (n=5)
P512C	0.019 ± 0.004 (n=4)	0.030 ± 0.006 (n=5)	1.77 ± 0.08 (n=5)	9.60 ± 0.47 (n=5)	16.8 ± 0.6 (n=5)
G513Y	0.041 ± 0.008 (n=5)	0.092 ± 0.023 (n=7)	2.33 ± 0.44 (n=6)	12.1 ± 1.7 (n=7)	12.4 ± 0.5 (n=5)
F515A	0.007 ± 0.002 (n=4)	0.025 ± 0.008 (n=8)	1.36 ± 0.04 (n=5)	5.20 ± 0.29 (n=8)	13.8 ± 0.8 (n=5)
D519A	0.034 ± 0.011 (n=5)	0.060 ± 0.020 (n=6)	2.23 ± 0.32 (n=6)	11.3 ± 1.5 (n=6)	13.4 ± 0.5 (n=6)
P520A	0.425 ± 0.067 (n=6)	0.396 ± 0.049 (n=8)	5.48 ± 1.15 (n=6)	27.8 ± 1.7 (n=8)	14.4 ± 0.6 (n=5)
P520G	0.294 ± 0.042 (n=5)	0.328 ± 0.061 (n=6)	5.45 ± 0.64 (n=4)	36.7 ± 3.4 (n=6)	--
S516A	0.077 ± 0.021 (n=4)	0.085 ± 0.014 (n=7)	4.40 ± 0.82 (n=7)	17.3 ± 1.6 (n=7)	19.1 ± 1.1 (n=6)
S516C	0.053 ± 0.014 (n=6)	0.095 ± 0.009(n=16)	2.47 ± 0.17 (n=12)	14.8 ± 1.0 (n=16)	16.0 ± 1.1 (n=7)
S516D	0.027 ± 0.006 (n=4)	0.030 ± 0.006 (n=6)	2.14 ± 0.17 (n=6)	10.4 ± 0.6 (n=6)	12.0 ± 0.7 (n=6)
S516E	0.015 ± 0.004 (n=4)	0.042 ± 0.008 (n=5)	1.39 ± 0.11 (n=5)	4.65 ± 0.46 (n=5)	11.8 ± 0.7 (n=5)
S516K	0.087 ± 0.020 (n=6)	0.119 ± 0.031 (n=7)	3.51 ± 0.43 (n=7)	22.3 ± 2.1 (n=7)	15.6 ± 0.8 (n=4)
S516N	0.452 ± 0.101 (n=5)	0.439 ± 0.069 (n=6)	9.00 ± 1.57 (n=5)	63.7 ± 10.5 (n=6)	20.4 ± 0.2 (n=5)
S516Q	0.048 ± 0.010 (n=6)	0.056 ± 0.008 (n=7)	2.10 ± 0.26 (n=7)	9.57 ± 0.66 (n=7)	14.3 ± 0.8 (n=6)
S516R	0.310 ± 0.034 (n=4)	0.309 ± 0.038 (n=8)	6.17 ± 0.60 (n=8)	38.4 ± 2.4 (n=8)	17.1 ± 0.8 (n=5)
S516T	0.069 ± 0.013 (n=5)	0.085 ± 0.010 (n=7)	2.78 ± 0.22 (n=7)	20.4 ± 1.7 (n=8)	17.3 ± 1.8 (n=4)
S516W	0.240 ± 0.047 (n=6)	0.242 ± 0.034 (n=8)	3.64 ± 0.56 (n=6)	20.0 ± 1.7 (n=8)	10.9 ± 0.6 (n=6)
S516Y	0.630 ± 0.035 (n=7)	0.632 ± 0.028 (n=9)	12.2 ± 3.9 (n=8)	33.9 ± 3.7 (n=9)	16.4 ± 1.5 (n=6)
F517A	0.007 ± 0.002 (n=8)	0.013 ± 0.002 (n=9)	1.17 ± 0.08 (n=6)	5.03 ± 0.09 (n=8)	12.4 ± 0.7 (n=5)
S615A	0.719 ± 0.149 (n=4)	0.674 ± 0.038 (n=7)	10.1 ± 1.9 (n=5)	40.4 ± 3.4 (n=6)	21.1 ± 1.6 (n=4)
S615C	0.533 ± 0.077 (n=4)	0.631 ± 0.123 (n=4)	7.91 ± 0.70 (n=4)	40.8 ± 7.2 (n=4)	19.1 ± 0.8 (n=3)
S615Y	0.325 ± 0.064 (n=5)	0.424 ± 0.058 (n=6)	5.13 ± 0.31 (n=6)	23.6 ± 1.6 (n=6)	19.5 ± 1.2 (n=4)
F623A	0.057 ± 0.021 (n=7)	0.130 ± 0.041 (n=12)	2.53 ± 0.64 (n=7)	6.88 ± 0.48 (n=12)	23.6 ± 2.1 (n=6)
S788A	0.051 ± 0.025 (n=4)	0.078 ± 0.031 (n=5)	2.26 ± 0.53 (n=5)	8.54 ± 0.59 (n=5)	25.0 ± 1.6 (n=5)
S791A	0.089 ± 0.024 (n=5)	0.114 ± 0.020 (n=7)	3.81 ± 0.52 (n=9)	14.8 ± 1.2 (n=7)	13.8 ± 1.0 (n=4)
S791C	0.346 ± 0.068 (n=5)	0.344 ± 0.031 (n=12)	4.71 ± 0.59 (n=8)	30.0 ± 3.1 (n=12)	25.6 ± 1.5 (n=5)
S791Y	0.009 ± 0.006 (n=4)	0.029 ± 0.014 (n=5)	1.36 ± 0.08 (n=4)	5.38 ± 0.23 (n=5)	16.9 ± 1.2 (n=5)

Presented are the fractions of non-desensitized receptors (I_{SS}/I_{Max} and I_{SS}/I_{Peak}) and the time constants of deactivation (τ_{Deact}), desensitization (τ_{Des}) and recovery from desensitization (τ_{RecDes}). The number of cells is given in parentheses. The values significantly different from the wild type values (t-Test, $p < 0.05$) are highlighted. Errors are SEMs.

Supplementary Movie Legends

Supplementary Movies 1-2. Ion permeation in the open state model. Visualization of the ion permeation through the open channel. Introduction of additional counter ions to the simulation to test for channel permeability is described in Methods. For clarity, only M2 and M3 helices are shown. The M2 and M3 helices are coloured green and yellow respectively; the permeating and non-permeating potassium ions are coloured red and purple, respectively. Suppl. Movie 1 starts at the 400 ns point in the Suppl. Fig. 1C; the length of the trajectory shown is 6 ns. Suppl. Movie 2 starts at the 520 ns point in the Suppl. Fig. 1C; the length of the trajectory shown is 20 ns. It is clear from the movies that one event of ion permeation occurs via exchange of the residing ion in the channel (Suppl. Movie 1), while the other one due to direct diffusion of the permeating ion (Suppl. Movie 2), an indicative of a fairly wide pore opening.

Supplementary Movies 3-6. **Animations of the principal component vectors PC1 and PC2**. PC1 (Suppl. Movies 3 and 4) and PC2 (Suppl. Movies 5 and 6) are shown for the TMD viewed extracellularly (Suppl. Movies 3 and 5) or parallel to the membrane (Suppl. Movies 4 and 6). In Suppl. Movies 4 and 6, two of four AMPA receptor subunits are shown, with the front and back subunits removed for clarity.
