# Supplementary figures and images for: Role of the Ion Channel Extracellular Collar in AMPA Receptor Gating
Source: Sci Rep. 2017 Apr 21;7:1050. doi: 10.1038/s41598-017-01146-z (PMC5430913; doi:10.1038/s41598-017-01146-z)

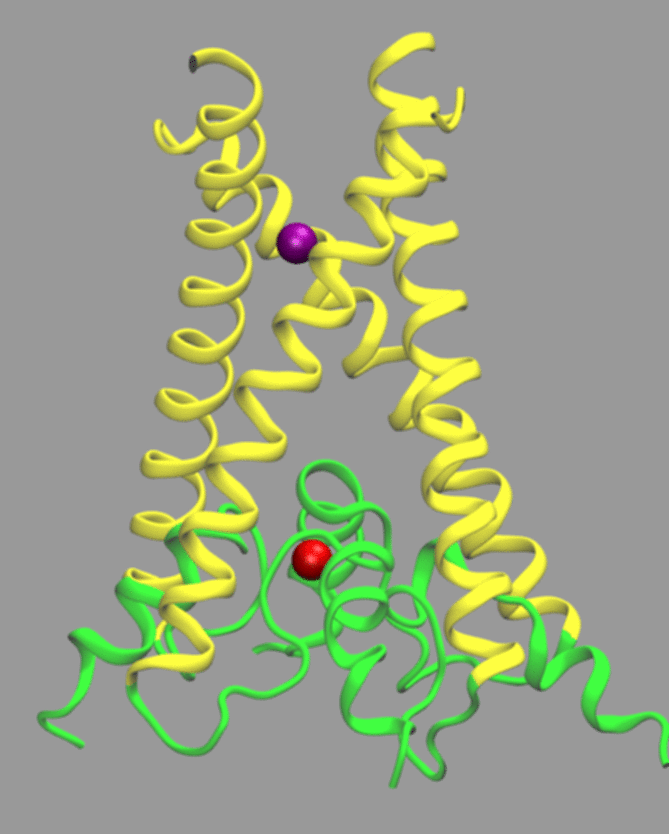

Supplement: Supplementary file 2 — Supplementary Movie 1 [file 41598_2017_1146_MOESM2_ESM.gif]

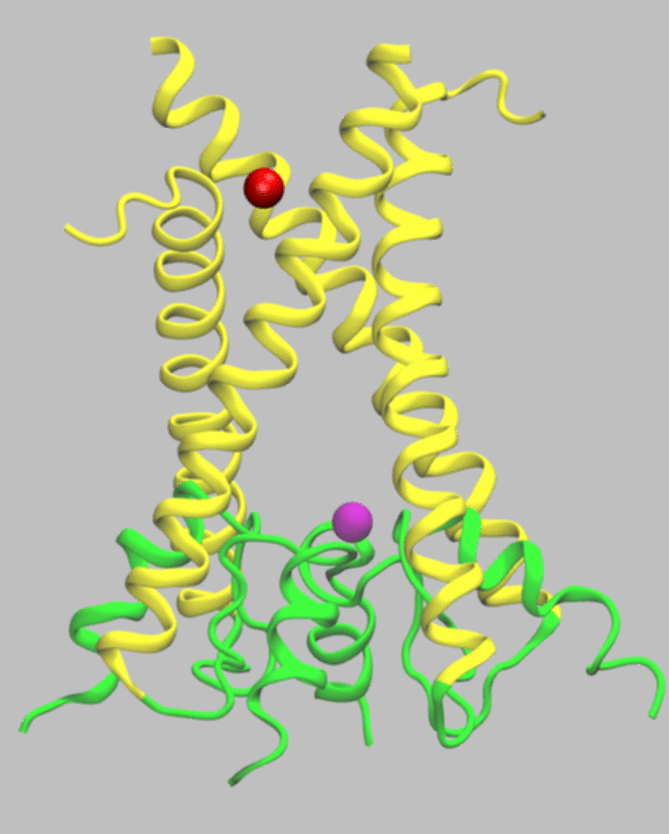

Supplement: Supplementary file 3 — Supplementary Movie 2 [file 41598_2017_1146_MOESM3_ESM.gif]

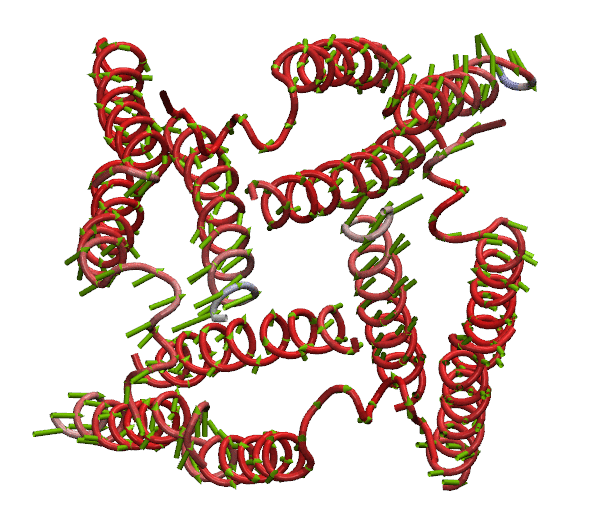

Supplement: Supplementary file 4 — Supplementary Movie 3 [file 41598_2017_1146_MOESM4_ESM.gif]

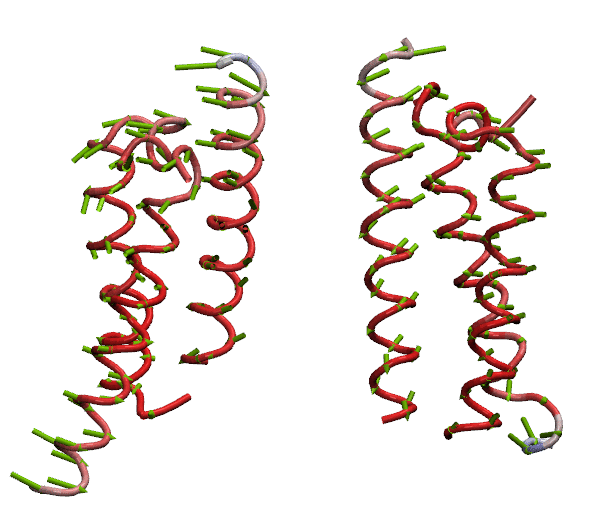

Supplement: Supplementary file 5 — Supplementary Movie 4 [file 41598_2017_1146_MOESM5_ESM.gif]

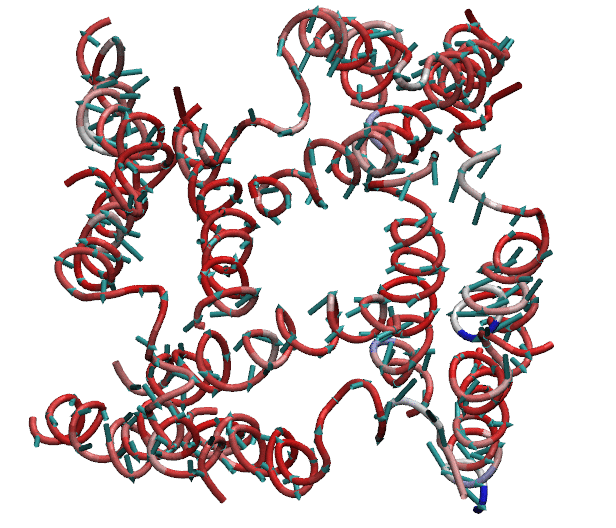

Supplement: Supplementary file 6 — Supplementary Movie 5 [file 41598_2017_1146_MOESM6_ESM.gif]

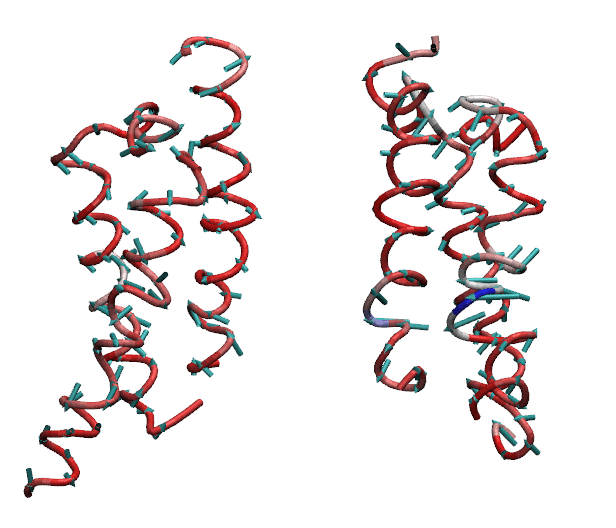

Supplement: Supplementary file 7 — Supplementary Movie 6 [file 41598_2017_1146_MOESM7_ESM.gif]
